# Association of Magnetoencephalographically Measured High-Frequency Oscillations in Visual Cortex With Circuit Dysfunctions in Local and Large-scale Networks During Emerging Psychosis

**DOI:** 10.1001/jamapsychiatry.2020.0284

**Published:** 2020-03-25

**Authors:** Tineke Grent-‘t-Jong, Ruchika Gajwani, Joachim Gross, Andrew I. Gumley, Rajeev Krishnadas, Stephen M. Lawrie, Matthias Schwannauer, Frauke Schultze-Lutter, Peter J. Uhlhaas

**Affiliations:** 1 Institute of Neuroscience and Psychology, University of Glasgow, Glasgow, Scotland; 2 Department of Child and Adolescent Psychiatry, Charité Universitätsmedizin, Berlin, Germany; 3 Mental Health and Wellbeing, Institute of Health and Wellbeing, University of Glasgow, Glasgow, Scotland; 4 Institute for Biomagnetism and Biosignalanalysis, University of Muenster, Muenster, Germany; 5 Department of Psychiatry, University of Edinburgh, Edinburgh, Scotland; 6 Department of Clinical Psychology, University of Edinburgh, Edinburgh, Scotland; 7 Department of Psychiatry and Psychotherapy, Medical Faculty, Heinrich Heine University, üsseldorf, Bergische Landstrasse 2, 40629 Düsseldorf, Germany

## Abstract

**Importance:**

Psychotic disorders are characterized by impairments in neural oscillations, but the nature of the deficit, the trajectory across illness stages, and functional relevance remain unclear.

**Objectives:**

To examine whether changes in spectral power, phase locking, and functional connectivity in visual cortex are present during emerging psychosis and whether these abnormalities are associated with clinical outcomes.

**Design, Setting, and Participants:**

In this cross-sectional study, participants meeting clinical high-risk criteria for psychosis, participants with first-episode psychosis, participants with affective disorders and substance abuse, and a group of control participants were recruited. Participants underwent measurements with magnetoencephalography and magnetic resonance imaging. Data analysis was carried out between 2018 and 2019.

**Main Outcomes and Measures:**

Magnetoencephalographical activity was examined in the 1- to 90-Hz frequency range in combination with source reconstruction during a visual grating task. Event-related fields, power modulation, intertrial phase consistency, and connectivity measures in visual and frontal cortices were associated with neuropsychological scores, psychosocial functioning, and clinical symptoms as well as persistence of subthreshold psychotic symptoms at 12 months.

**Results:**

The study participants included those meeting clinical high-risk criteria for psychosis (n = 119; mean [SD] age, 22 [4.4] years; 32 men), 26 patients with first-episode psychosis (mean [SD] age, 24 [4.2] years; 16 men), 38 participants with affective disorders and substance abuse (mean [SD] age, 23 [4.7] years; 11 men), and 49 control participants (mean age [SD], 23 [3.6] years; 16 men). Clinical high-risk participants and patients with first-episode psychosis were characterized by reduced phase consistency of β/γ-band oscillations in visual cortex (*d* = 0.63/*d* = 0.93). Moreover, the first-episode psychosis group was also characterized by reduced occipital γ-band power (*d* = 1.14) and altered visual cortex connectivity (*d* = 0.74-0.84). Impaired fronto-occipital connectivity was present in both clinical high-risk participants (*d* = 0.54) and patients with first-episode psychosis (*d* = 0.84). Importantly, reductions in intertrial phase coherence predicted persistence of subthreshold psychosis in clinical high-risk participants (receiver operating characteristic area under curve = 0.728; 95% CI, 0.612-0.841; *P* = .001).

**Conclusions and Relevance:**

High-frequency oscillations are impaired in the visual cortex during emerging psychosis and may be linked to behavioral and clinical impairments. Impaired phase consistency of γ-band oscillations was also associated with the persistence of subthreshold psychosis, suggesting that magnetoencephalographical measured neural oscillations could constitute a biomarker for clinical staging of emerging psychosis.

## Key Points

**Question:**

Are high-frequency oscillations in visual cortex impaired during early stages of psychosis?

**Findings:**

In this cross-sectional study, there were significant impairments in the variability, power, and connectivity of neural oscillations during visual processing in clinical high-risk participants and patients with first-episode psychosis that were associated with impaired functioning and cognitive deficits. Moreover, the increased variability of γ-band oscillations in visual cortex was also associated with the persistence of subthreshold psychotic symptoms in clinical high-risk participants.

**Meaning:**

Impaired high-frequency oscillations in visual cortex are an important aspect of circuit dysfunction, which could constitute a biomarker for clinical staging of emerging psychosis.

## Teaser

This study examines whether changes in spectral power, phase locking, and functional connectivity in visual cortex are present during emerging psychosis and whether these abnormalities are associated with clinical outcomes.

## Introduction

Neural oscillations are a crucial aspect of normal brain functioning owing to their role in facilitating communication between neuronal populations,^1^ a process that is closely linked to the integrity of sensory and cognitive processes.^2,3^ There is emerging evidence that psychotic disorders with pronounced cognitive impairments, such as schizophrenia, involve aberrant neuronal oscillations,^4^ but the nature of the impairment, the onset of deficits, and clinical relevance remain unclear.

β/γ-Band oscillations^5,6,7^ but also lower frequencies are impaired during sensory^8^ and cognitive tasks^9^ in schizophrenia.^10,11^ During normal brain functioning, inhibition of excitatory pyramidal cells through different classes of aminobutyric acid (GABA)ergic interneurons lead to the emergence of neural oscillations.^12,13,14,15^ Converging evidence from genetics,^16^ postmortem data,^17,18^ and brain imaging have^19^ highlighted that GABAergic as well as glutamatergic neurotransmission is impaired in schizophrenia, supporting the possibility that measurements with electro/magnetoencephalography (EEG/MEG) could be important for translational research aimed at identifying circuit mechanisms in the disorder.^10^

Critical questions concerning the role of neural oscillations in the pathophysiology of schizophrenia are the onset of abnormalities, the nature of the deficit, and functional relevance. Early signs of psychosis as well as associated cognitive deficits are already present several years prior to the full emergence of schizophrenia,^20^ and thus, research efforts have shifted the focus toward identifying circuit abnormalities and biomarkers in participants who are at risk for the development of psychotic disorders that could allow for early intervention and clinical staging.^21,22^

There is only limited evidence available on alterations of neural oscillations in individuals meeting clinical high-risk criteria for psychosis (CHR-P).^23,24^ To address this fundamental question, we applied a state-of-the-art MEG approach to examine low-frequency and high-frequency oscillations during a visual paradigm in CHR-P participants, patients with first-episode psychosis (FEP), and participants with substance-related and affective disorders. Magnetoencephalography is characterized by an improved signal-to-noise ratio for measurements of high-frequency oscillations compared with EEG^25,26^ and is ideally suited for source reconstruction, allowing the identification of anatomical layout of generators with high spatial resolution.^27^

Based on models of developing psychosis that have highlighted the central role of visual deficits during the early stages of psychosis^28,29^ that predict transition to psychosis^30^ as well as the importance of high-frequency oscillations for the integrity of visual perception,^1,31,32^ we predicted that CHR-P participants would be characterized by a circumscribed dysfunction of β/γ-band oscillations in visual cortex that would be linked to clinical outcomes. Specifically, we focused on the persistence of attenuated psychotic symptoms (APS) because there is evidence to suggest that persistent APS are associated with poor outcomes^33^ and cognitive deficits in CHR-P populations.^34^ Patients with FEP, on the other hand, would involve large-scale dysfunctions of induced oscillations and effective connectivity between frontal and visual areas, consistent with a disconnection syndrome.^35,36^

## Methods

### Participants

Four groups of participants (total n = 232) were recruited: (1) participants meeting CHR-P criteria (n = 119) from the ongoing Youth Mental Health Risk and Resilience (YouR) Study^37^; (2) 38 participants who did not meet CHR-P criteria (CHR-N) and were characterized by nonpsychotic disorders, such as affective disorders (n = 11), anxiety disorders (n = 16), eating disorders (n = 1), and/or substance abuse (n = 10); (3) 26 patients with FEP (13 antipsychotic-naive); and (4) 49 healthy control individuals (HC) without an axis I diagnosis or family history of psychotic disorders. Data from 10 patients with FEP and 10 HC have been published previously.^38^

The CHR-P status was confirmed by ultrahigh-risk criteria according to the Comprehensive Assessment of At Risk Mental States (CAARMS) interview^39^ and the Cognitive Disturbances and Cognitive-Perceptive Basic Symptoms criteria according to the Schizophrenia Proneness Instrument, Adult version (SPI-A)^40^ (see Uhlhaas et al^37^). Patients with FEP were assessed with the Structured Clinical Interview for *DSM-IV* (Table)^41^ and with the Positive and Negative Symptom Scale.^42^ For all groups except patients with FEP, neurocognition was assessed with the Brief Assessment of Cognition in Schizophrenia (BACS).^43^ The study was approved by the ethical committees of University of Glasgow and the National Health Services Research Ethical Committee Glasgow and Greater Clyde. All participants provided written informed consent.

**Table.** Demographics, Clinical Data, and Task Performance

Demographic	HC	CHR-N	CHR-P	FEP	Group effect^a^	Pairwise comparisons
No. of participants	49	38	119	26	NA	NA
Age, (SD), y	23 (3.6)	23 (4.7)	22 (4.4)	24 (4.2)	NA	NA
Male/female sex, No. (% male)	16/33 (32.7)	11/27 (28.9)	32/87 (26.9)	16/10 (61.5)	χ^2^_3_ = 11.9; *P* = .008	FEP to HC: *P* = .016 ; FEP to CHR-N: *P* = .001; FEP to CHR-P: *P* = .01
Education, mean (SD), y	17 (3.0)	16 (3.5)	15 (3.1)	15 (3.0)	*F*_3,76_ = 3.5; *P* = .02	CHR-P to HC: *P* = .03
BACS,^b^ mean (SD)						
Verbal memory	52 (8.7)	0.01 (1.1)	−0.36 (1.3)	NA	NA	NA
Digit sequencing	21 (2.1)	0.14 (1.2)	−0.15 (1.5)	NA	NA	NA
Token motor	81 (11.6)	−0.66 (1.1)	−0.98 (1.3)	-	*F*_2,93_ = 13.8; *P* < .001	CHR-N to HC: *P* = .01; CHR-P - HC: *P* < .001
Verbal fluency	59 (13.9)	−0.22 (1.0)	0.05 (1.3)	NA	NA	NA
Symbol coding	74 (11.8)	0.00 (1.3)	−0.58 (1.1)	NA	*F*_2,84_ = 6.8; *P* = .002	CHR-P - HC: *P* = .004; CHR-P – CHR-N: *P* = .04
Tower of London	19 (1.7)	0.15 (1.3)	−0.21 (1.5)	NA	NA	NA
Composite score	304 (24.2)	−0.15 (1.2)	−0.63 (1.4)	NA	*F*_2,93_ = 5.8; *P* = .004	CHR-P - HC: *P* = .004
CAARMS, mean (SD)						
Unusual thought content	NA	1 (1.2)	2 (1.9)	NA	NA	NA
Nonbizarre ideas	NA	1 (1.1)	3 (1.8)	NA	NA	NA
Perceptual abnormalities	NA	1 (1.3)	3 (1.6)	NA	NA	NA
Disorganized speech	NA	1 (0.9)	1 (1.4)	NA	NA	NA
Total severity score	NA	6 (6.1)	29 (17.8)	NA	NA	NA
GAF, mean (SD)	88 (6.4)	70 (12.8)	57 (13.4)	41 (16.9)	*F*_3,75_ = 167; *P* < .001	All contrasts *P* < .001
GF-role, mean (SD)	8.6 (0.8)	8.1 (0.8)	7.4 (1.2)	NA	*F*_2,99_ = 29.6; *P* < .001	CHR-N - HC: *P* = .037; CHR-P to HC: *P* < .001; CHR-P to CHR-N: *P* < .001
GF-social, mean (SD)	8.8 (0.4)	8.2 (0.8)	7.5 (1.2)	NA	*F*_2,94_ = 59.5, *P* < .001	CHR-N - HC: *P* < .001; CHR-P to HC: *P* < .001; CHR-P to CHR-N: *P* < .001
PANSS, mean (SD)						
Positive	NA	NA	NA	18 (7.2)	NA	NA
Negative	NA	NA	NA	15 (9.3)	NA	NA
Cognitive	NA	NA	NA	20 (9.2)	NA	NA
Excitement	NA	NA	NA	9 (4.3)	NA	NA
Depression	NA	NA	NA	12 (5.9)	NA	NA
Total score	NA	NA	NA	74 (28.4)	NA	NA
Medication, No. (%)^c^						
None	48	27	61	6	NA	NA
Antidepressants	0	11	47	13	NA	NA
Mood stabilizers	0	0	5	0	NA	NA
Antipsychotics	0	0	3	13	NA	NA
Other (unknown)	1 (0)	2 (0)	21 (0)	5 (0)	NA	NA
CHR-P categories						
SPI-A (COGDIS/COPER/both items)	NA	NA	30 (4/15/11)	NA	NA	NA
CAARMS (APS/vulnerability criteria)	NA	NA	89 (87/2)	NA	NA	NA
CAARMS plus SPI-A (COGDIS/COPER/both items)	NA	NA	55 (9/22/24)	NA	NA	NA
MINI categories						
Depressive/mood disorders	NA	11	75	NA	NA	NA
Anxiety disorders/posttraumatic stress disorder/obsessive-compulsive disorder	NA	16	87	NA	NA	NA
Drug/alcohol abuse/dependence	NA	10	42	NA	NA	NA
Eating disorders	NA	1	10	NA	NA	NA
*DSM-IV*/Structured Clinical Interview						
Schizophrenia	NA	NA	NA	9	NA	NA
Schizophreniform disorder	NA	NA	NA	3	NA	NA
Schizoaffective disorder	NA	NA	NA	1	NA	NA
Psychotic disorder NOS	NA	NA	NA	8	NA	NA
Brief psychotic disorder	NA	NA	NA	1	NA	NA
Mood disorders with psychotic features	NA	NA	NA	4	NA	NA
Trial No., total included (SD)	197 (16.3)	194 (16.2)	185 (26.5)	181 (29.2)	*F*_3,80_ = 5.9; *P* = .001	CHR-P to HC: *P* = .002
Task performance						
Accuracy, % correct (SD)	92.2 (6.9)	92.0 (5.6)	88.0 (9.8)	85.9 (12.8)	*F*_3,79_ = 5.5, *P* = .002	CHR-P to HC: *P* = .01; CHR-P vs CHR-N: *P* = .01
Reaction time, mean (SD), ms	528 (68.4)	524 (80.1)	545 (84.9)	577 (100.1)	NA	NA
Response variance,^d^ mean (SD), ms	151.7 (37.5)	154.3 (34.9)	164.3 (42.2)	179.1 (40.8)	*F*_3,79_ = 3.3; *P* = .02	FEP to HC: *P* = .03

Abbreviations: APS, attenuated psychotic symptoms; BACS, Brief Assessment of Cognition in Schizophrenia; CAARMS, Comprehensive Assessment of At Risk Mental States; CHR-N, clinical high risk negative; CHR-P, clinical high risk positive; COGDIS/COPER, Cognitive Disturbances and Cognitive-Perceptive Basic Symptoms criteria; FEP, first-episode psychosis; GAF, global assessment of functioning; GF, global functioning; HC, healthy control individual; MINI, Mini-International Neuropsychiatric Interview; PANSS, Positive and Negative Symptom Scale; SPI-A, Schizophrenia Proneness Instrument, Adult version.

^a^ All *F* tests are Welch based; α = .05, 2-sided, 1000 samples bootstrapping, post hoc Games-Howell correction for type I errors.

^b^ BACS scores for clinical groups were standardized to control group data, controlled for sex category.

^c^ If multiple medications were reported, they were scored separately in the different categories listed.

^d^ Response variance equals standard deviation of response times across trials.

### Clinical Follow-up

Participants meeting CHR-P and CHR-N criteria were reassessed at 3-, 6-, 9-, 12-, 18-, 24-, 30-, and 36-month intervals to examine persistence of CHR-P criteria and transition to psychosis (eMethods in the Supplement).

### Stimuli and Task

**Figure 1.**
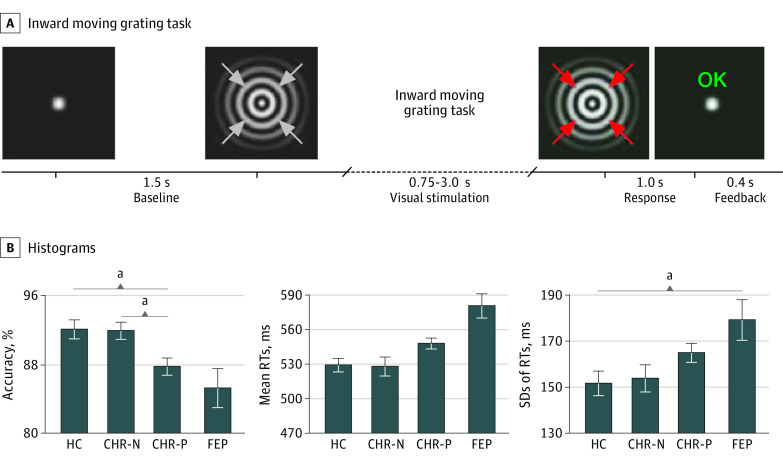
Paradigm and Task Performance A, Inward moving grating task: participants report, by button press, the onset of a change in velocity of inward motion of the visual stimulus (correct response window, 200-1200 milliseconds). Feedback on performance was provided on every trial, shortly after the response onset terminated stimulus presentation. B, Histograms of group means and standard errors for accuracy (% correct), mean reaction times (RTs), and behavioral variability (intraindividual standard deviation of RTs). CHR-N indicates clinical high risk negative; CHR-P, clinical high risk positive; FEP, first-episode psychosis; HC, healthy control individuals. ^a^Indicates significant group differences (Welch *F* tests, α = .05, 2-sided, 1000 samples bootstrapping, Games-Howell corrected for multiple comparisons).

Participants were presented with 3 blocks of 80 trials, with each trial consisting of a circular sinewave grating that contracted toward central fixation.^44^ The task of the participants was to detect and respond by button press to a velocity increase of the stimulus, randomly occurring between 750 and 3000 milliseconds (Figure 1).

### Neuroimaging

Magnetoencephalography data were acquired using a 248-channel 4D-BTI magnetometer system (MAGNES 3600 WH, 4-dimensional neuroimaging; Bio-Medicine), recorded with 1017.25-Hz sampling rate and DC-400 Hz online filtered. T1 anatomical scans (3-dimensional magnetization-prepared rapid gradient-echo sequences) were collected for patient-specific source localization of MEG activity (eMethods in the Supplement).

### Magnetoencephalography Data Analysis

Magnetoencephalography data were analyzed with MATLAB using the open-source Fieldtrip Toolbox.^45^ Preprocessing included correct trials only with nonoverlapping 3.8-second segments (1-second baseline), time locked to the onset of the visual grating. Line noise was attenuated with a discrete 50-Hz Fourier transform filter, and faulty sensors with large signal variance or flat signals were removed. Data were denoised relative to MEG reference channels and downsampled to 300 Hz. Artifact-free data were created by removing trials with excessive transient muscle activity, slow drift, or superconducting quantum interference device jumps using visual inspection, and independent component analysis–based removal of eye blink, eye movement, and electrocardiographic artifacts. Data were then submitted to time-frequency (TFR) analyses (1-90 Hz, stepsize 50 milliseconds, 450 milliseconds sliding-window fast-fourier transformed [FFT]; Hanning tapered), computed for planar-orientation transformed MEG data.^46^

**Figure 2.**
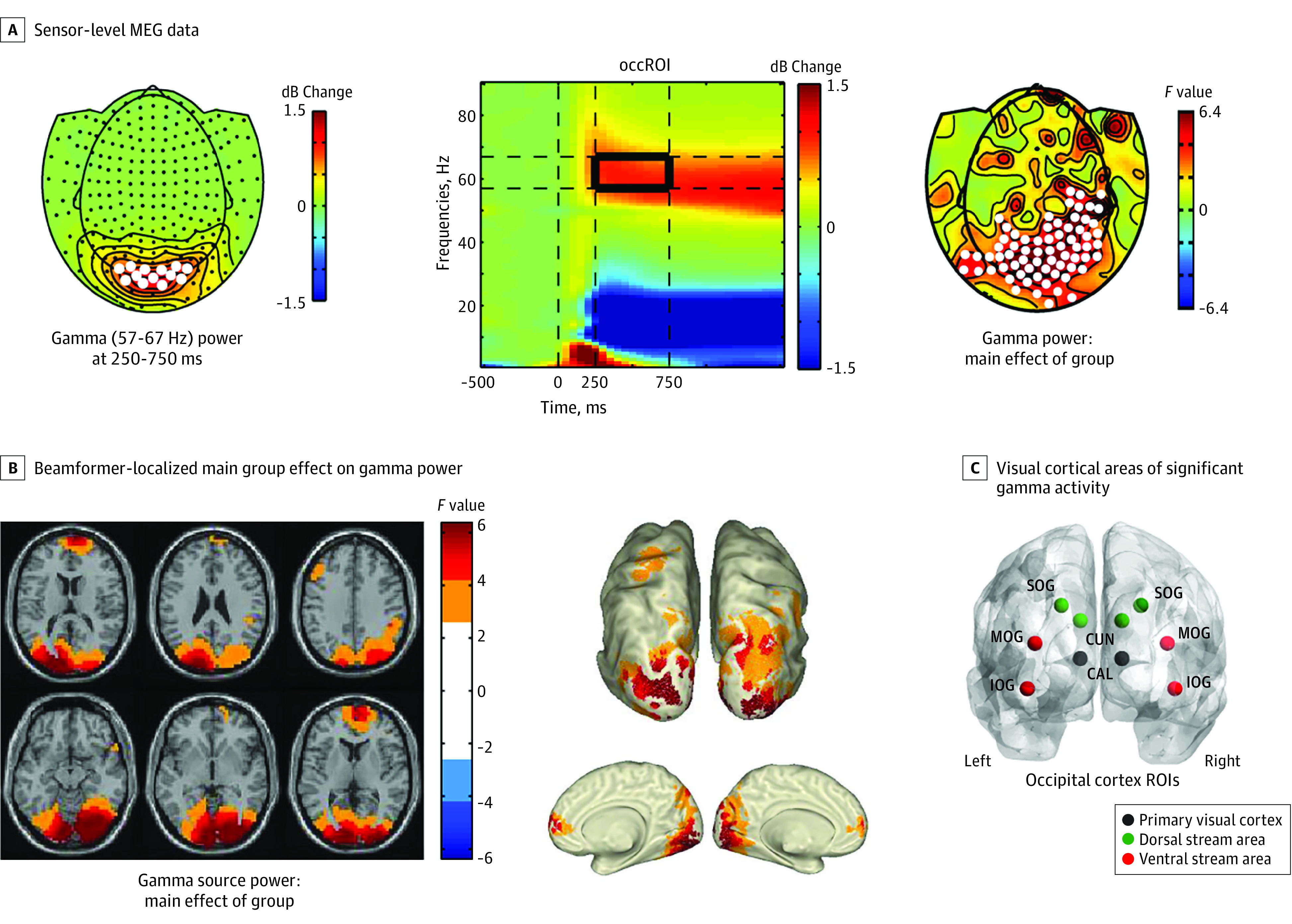
Sensor and Source-Power Magnetoencephalography (MEG) Data A, Sensor-level MEG data: left topographical distribution plot shows grand average–induced γ power (n = 232) changes from baseline, with white dots marking the sensors for which the time frequency response (TFR) plot in the middle is plotted. In the TFR plot, the outlined (black box) window indicates the window of statistical testing for group differences in γ power. The TFR plot shows evoked activity from stimulus onset (time zero) to approximately 250 milliseconds, from which latency-induced activity is shown up to 1500 milliseconds. Right topographical distribution plot shows *F* values of significant (marked with white dots) sensors showing a main group effect on γ power. B, Beamformer-localized main group effect on γ power. Lighter-blue and light-orange values mark areas of significant effects uncorrected for multiple comparisons, whereas the darker colors display false discovery rate–corrected areas (α = .05; 2-sided). C, Locations across visual cortical areas of significant γ activity from which virtual channel time-series MEG data were reconstructed. The 10 occipital regions of interest (ROIs) included 3 subregions covering the primary visual cortex. CAL indicates calcarine; CUN, cuneus; IOG, inferior occipital gyrus; MOG, middle occipital gyrus; SOG, superior occipital gyrus.

**Figure 3.**
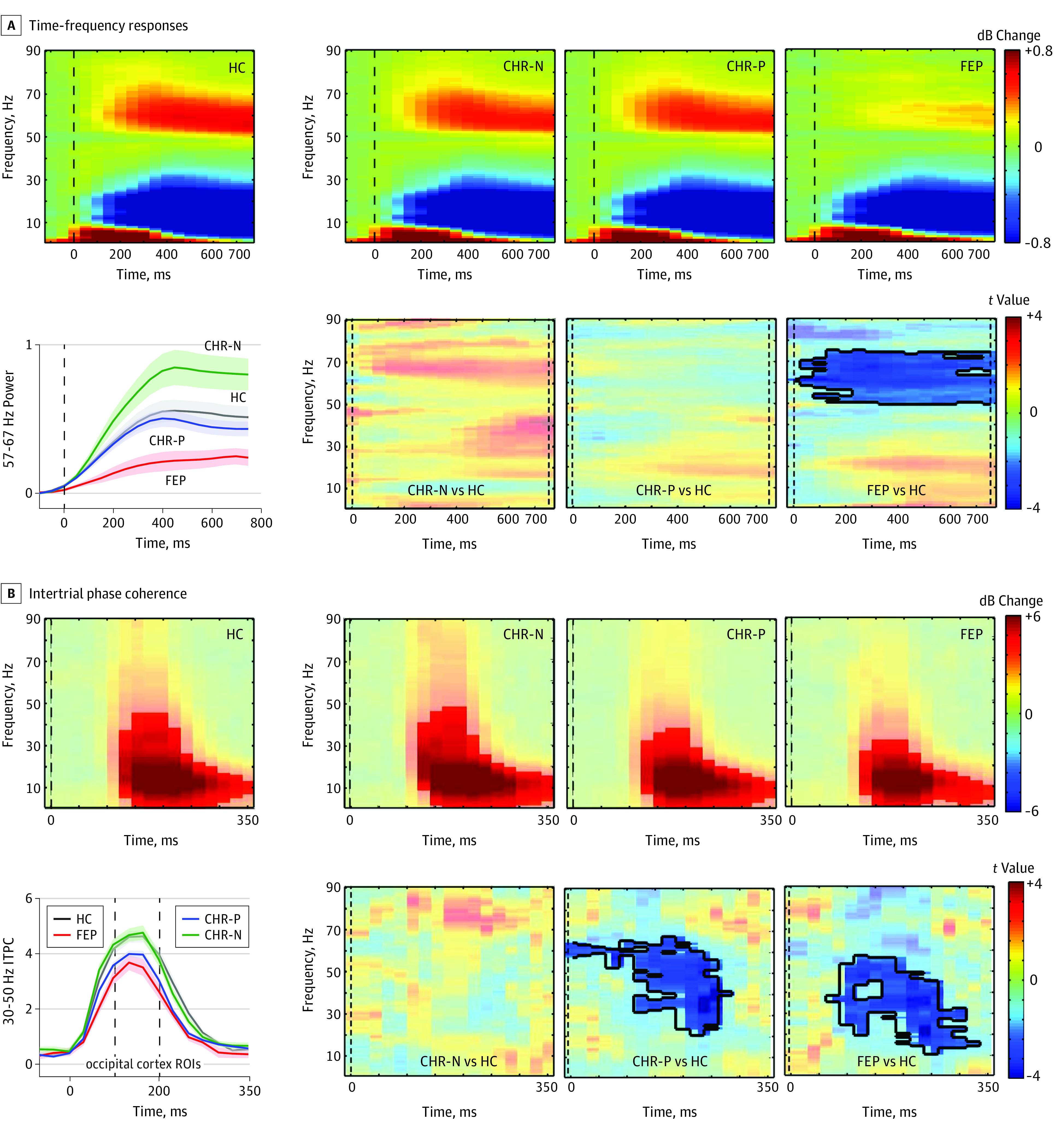
Virtual Channel Time Frequency Response (TFR) and Intertrial Phase Coherence (ITPC) Analyses A, Top 4 panels show per group the TFR, averaged over all virtual-channel regions of interest (ROIs) shown in Figure 2C. Bottom right panels: TFR plot with statistical results (nonparametric, Monte Carlo–based permutation independent *t* tests) of group differences in time-frequency clusters between 0 and 750 milliseconds, with significant clusters outlined and the remaining nonsignificant time-frequency bins masked out (opacity, 0.45). The line graph on the left shows the γ (57-67 Hz) response over time per group, with error bars representing standard error of the mean. B, Top panels show the ITPC responses per group and bottom panels show the significant group differences (between 0-350 milliseconds), with significant time-frequency bins outlined. The line graph on the left shows γ (30-50 Hz) range ITPC responses per group, with error bars representing standard error of the mean. CHR-N indicates clinical high risk negative; CHR-P, clinical high risk positive; FEP, first-episode psychosis; HC, healthy control individuals.

Whole-brain source estimation of γ-band power (57-67 Hz) between 250 and 750 milliseconds was computed using the Dynamic Imaging of Coherent Sources beamforming approach^47^ (eMethods in the Supplement). γ-Band source data were statistically tested across groups to determine the location of main effects (Figure 2). These were then used to guide selection of the main regions of interest (ROIs) for more fine-grained virtual channel analyses (Figure 2 and Figure 3C) (eMethods in the Supplement). Virtual channel time series were used for the analysis of event-related fields (ERF), TFR, inter-trial phase coherence (ITPC),^48^ baseline FFT, and Granger causality (GC).

Granger-causality estimates were computed using a nonparametric approach, including spectral density matrices estimated directly from FFT-data (250-750 milliseconds; DC-149 Hz; Hanning tapered; 5-Hz frequency smoothing; 1-Hz resolution; data zero-padded to 4 seconds), followed by matrix factorization and variance decomposition. Epochs were split into 2 × 250-millisecond segments to increase trial numbers (see Michalareas et al^32^). The middle occipital gyrus and cuneus ROIs were not used in the GC analyses to minimize overlap between primary visual, dorsal, and ventral stream connectivity estimates. Granger-causality data from each pair were averaged over hemisphere to create 4 main ROI pairs for statistical testing. To determine the alterations in feedforward (FF) vs feedback (FB) GC activity, we also computed the directed asymmetry index (DAI; see Michalareas et al,^32^ Bastos et al,^49^ and eMethods in the Supplement).

### Statistical Analysis

Group differences in trial numbers, γ-band peak frequency, behavioral performance, demographic, and clinical data were assessed with 1-way Welch analysis of variance (ANOVA); 2-sided α level of .05. Brief Assessment of Cognition in Schizophrenia data were first *z*-normalized to the HC data. Bootstrapping (n = 1000) and Games-Howell correction were used to control type I errors in post hoc pairwise group comparisons.

Statistical testing of group differences in MEG virtual-channel data included nonparametric Monte-Carlo–based permutation (n = 2000) independent *F* test (main group effect) and post hoc *t* test statistics^45^ for ERFs (0-750 milliseconds); TFRs and ITPC power (1-90 Hz, 0-750 milliseconds for TFR power, 0-350 milliseconds for ITPC, and dB change from a 500-millisecond baseline); baseline FFT spectra (1-90 Hz); and GC data. Type I errors were controlled by cluster statistics across time and/or frequency (eMethods in the Supplement). Finally, binary logistic regression and receiver operating characteristic curve (ROC) analyses were used to examine the association between MEG parameters and clinical outcomes in CHR-P participants (eMethods in the Supplement).

## Results

### Demographic Data

The FEP group included significantly more men than the HC (χ^2^_1_ = 5.8; *P* = .02), CHR-N (χ^2^_1_ = 6.7; *P* = .01), and CHR-P (χ^2^_1_ = 11.6, *P* = .001) groups (Table). The BACS composite score was significantly reduced in CHR-P participants compared with HC (−0.84; 95% CI, −1.43 to −0.25; *P* = .004). All clinical groups differed from HC in global assessment of functioning (GAF) scores (CHR-N, 17.7; 95% CI, 11.7 to 23.8; *P* < .001; CHR-*P*, 30.3; 95% CI, 26.3 to 34.2; *P* < .001; FEP, 46.6, 95% CI, 37.2 to 56.0; *P* < .001). Both CHR-P and CHR-N groups also differed from HC in global role and social functioning (CHR-N, −0.63; 95% CI, −0.28 to −0.99; *P* < .001; CHR-*P*,  −1.35; 95% CI, −1.05 to −1.66; *P* < .001).

### Follow-up Outcomes

We examined persistence of APS up to 12 months in CHR-P participants who met APS criteria at baseline (n = 84). For 75 CHR-P participants, at least 1 follow-up assessment was available. Thirty-nine CHR-P participants continued to meet APS criteria (APS-persistent group) while 36 CHR-P participants were characterized by a remission of APS-criteria (eResults in the Supplement). Moreover, 9 of 119 CHR-P participants made a transition to psychosis (mean follow-up period, 17.3 months). Eight transitions occurred in the APS-persistent group.

### Task Performance

The CHR-P group was characterized by reduced response accuracy (−4.2%; 95% CI, −7.6 to −0.7; *P* = .01), while the patients with FEP were significantly more variable in reaction times (RTs) (27 milliseconds; 95% CI, 2 to 53; *P* = .03) compared with HC (Table).

### Sensor-Level Analysis

Modulation of spectral power was characterized by early evoked activity (<approximately 250 milliseconds), which is phase locked and time locked to the onset of the stimulus and sustained induced activity that represents non–phase-locked oscillations (>250 milliseconds) (Figure 2). Task-induced γ peak frequency across participants was approximately 62 Hz. A main group effect (cluster *F*_3,228_ = 341.7; *P* < .001; 95% CI range, −0.0004 to 0.002) for 57- to 67-Hz power was found over occipital and parietal-temporal regions (Figure 2A), with no differences in any other frequency range. Post hoc test results revealed significantly increased γ power for CHR-N vs HC over superior occipital-parietal regions (cluster *t*_85_ = 48.4; *P* = .03; 95% CI range, 0.0147-0.0453) and significantly decreased γ power (cluster *t*_73_ = −50.7; *P* = .02; 95% CI range = 0.0143-0.0257) over inferior occipital regions for FEP compared with HC (eFigure 1 in the Supplement).

### Virtual Channel Analyses: TFR and ITPC Analyses

A cluster of sustained γ-band power decreases across all visual cortex ROIs (Figure 3A) for the FEP group compared with HC (TFR cluster approximately 50-75 Hz; approximately 0-750 milliseconds; cluster *t*_73_ = −791.8; *P* = .007; 95% CI range, 0.0033-0.0107) were observed in primary visual cortex as well as in ventral stream areas (eFigure 2A in the Supplement). The CHR-N and CHR-P groups did not show spectral power changes in any frequency range.

Differences in β/γ-band ITPC values were found for both CHR-P and FEP participants compared with HC (CHR-P: TFR-cluster approximately 21-68 Hz; approximately 125-275 milliseconds; cluster *t*_166_ = −509.1; *P* = .005; 95% CI range, 0.0022 to 0.0078; FEP: TFR-cluster approximately 11-57 Hz; approximately 75-325 milliseconds; cluster *t*_73_ = −633.1; *P* = .002; 95% CI, 0.0018-0.0022) (Figure 3B) that involved primary visual as well as ventral stream regions and that extended to dorsal stream areas in the patients with FEP (eFigure 2B in the Supplement). The CHR-N group showed intact ITPC spectral power across all visual ROIs and frequencies.

### Behavioral and Magnetoencephalographical Parameters Associated With APS Persistence in the CHR-P Group

**Figure 4.**
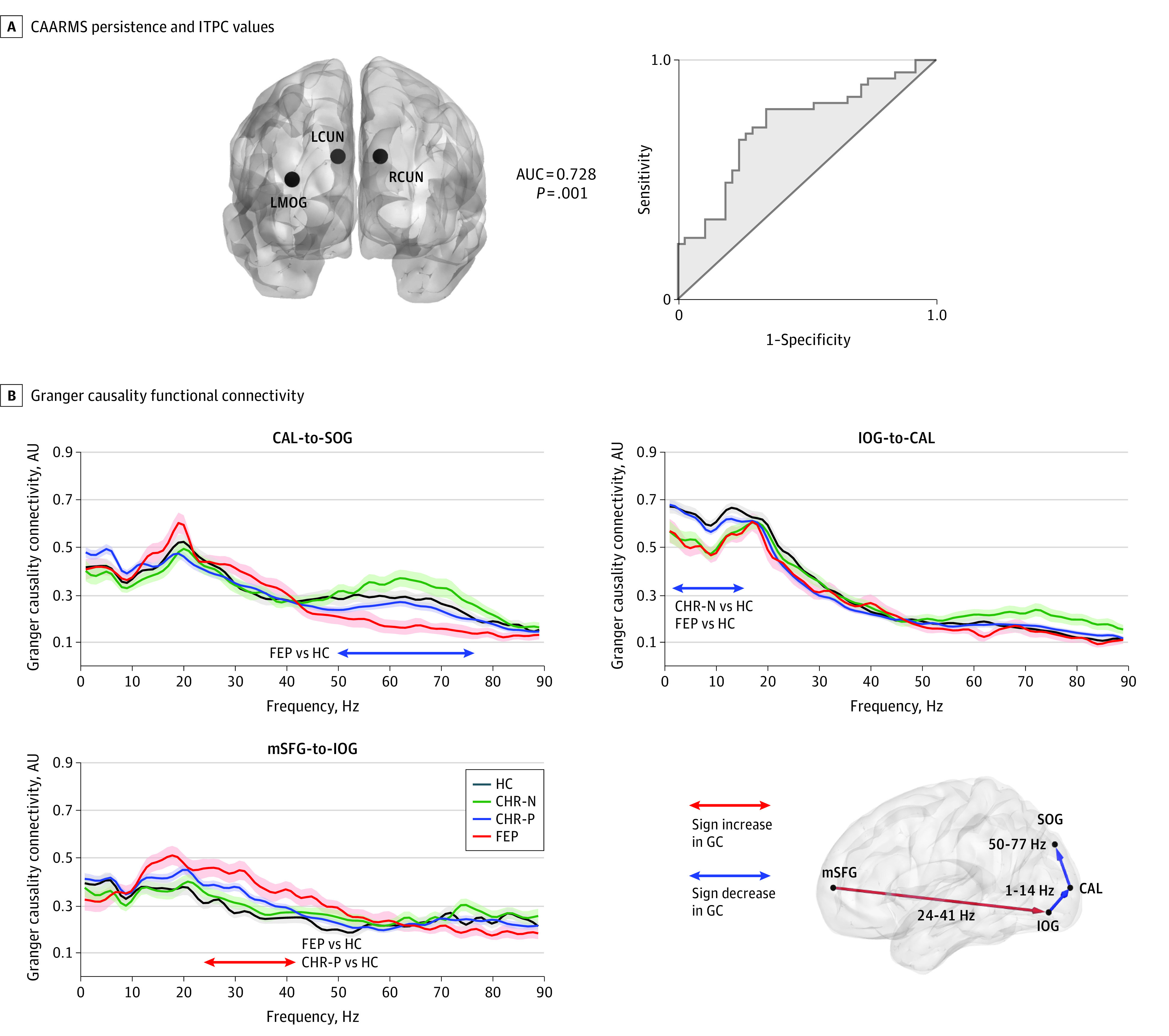
Receiver Operating Characteristic (ROC) Curve Analysis and Granger Causality (GC) Functional Connectivity A, On the right, ROC curve computed from prediction probabilities associated with a significant logistic regression model for predicting 12 months Comprehensive Assessment of At Risk Mental States (CAARMS; attenuated psychotic symptoms) persistence status from baseline magnetoencephalography (MEG) recordings of intertrial phase coherence (ITPC) of visual cortex responses (left and right cuneus and left middle occipital gyrus; locations shown in left panel). B, Results of cluster-based statistics on GC data showing range of significant effects in first-episode psychosis (FEP), clinical high-risk positive (CHR-P), and clinical high risk negative (CHR-N) groups, compared with healthy control individuals (HC). The main connections tested are plotted on a smoothed surface of a standard Montreal Neurological Institute brain, with red lines representing increased and blue lines decreased GC values, compared with HC. For each significant connection, GC values are plotted across the frequency spectrum, separately per group (with error bars indicating standard error of the mean), and a horizontal line indicating the frequency range of significant group effects. The GC was computed for data between 250 to 750 milliseconds after stimulus onset. The directed asymmetry indices were all positive in the significant contrasts, indicating feed-forward flow of information between the nodes. AUC indicates area under curve.

Intertrial phase coherence data (30-50 Hz; 125-200 milliseconds) from 10 occipital ROIs, accuracy, RTs, and RT variability were entered into a regression model to predict persistence of APS criteria in the CHR-P group. Only γ-band ITPC (30-50 Hz) activity contributed significantly to the model. Specifically, ITPC data from the left/right cuneus and left middle occipital gyrus ROIs led to a significant model (χ^2^_3_ = 14.4; *P* = .002) that explained 22.2% of the variance (Nagelkerke *R*^2^ = 0.222). The associated ROC curve was also significant (Figure 4A: area under the curve, 0.728; 95% CI, 0.612-0.841; *P* = .001) (eMethods and eResults in the Supplement).

### Regions of Interest: Baseline Power Spectra and ERF Responses

No group differences in baseline spectral power (1-90 Hz) or ERF amplitudes were observed in any visual cortex ROI (eFigures 3 and 4 in the Supplement).

### GC Connectivity

A main group effect was found for 2 connections in visual cortex (Figure 4B: calcarine [CAL] to superior occipital gyrus; approximately 50-77 Hz; cluster *P* = .004; 95% CI range, 0.0014-0.0066; inferior occipital gyrus [IOG] to CAL: approximately 1-14 Hz; cluster *P* = .02; 95% CI range, 0.0163-0.0237) and a fronto-occipital connection (medial-superior frontal [mSFG] to IOG: approximately 24-41 Hz; cluster *P* = .007; 95% CI range, 0.0033 to 0.0107). Post hoc comparisons revealed decreased connectivity in the FEP group in visual cortex (CAL to superior occipital gyrus: DAI = 0.04; *t*_73_ = −3.0; *P* = .006; 95% CI range, 0.0026 to 0.0094; IOG to CAL: DAI = 0.11; *t*_73_= −3.5; *P* = .004; 95% CI range, 0.0012-0.0068) but increased fronto-occipital connectivity (mSFG-to-IOG connection: DAI = 0.11; *t*_73_ = 4.5; *P* < .001; 95% CI range, −0.0004 to 0.002). Comparable long-range connectivity changes were seen in the CHR-P group (mSFG-to-IOG: DAI = 0.07;* t*_166_ = 3.19; *P* = .003; 95% CI range, 0.0028-0.0032). The CHR-N group showed decreased IOG-to-CAL connectivity (DAI = 0.11;* t*_85_ = −3.5; *P* = .002; 95% CI range, 0.0002-0.0038).

### Correlations

Correlations were tested using linear regression models (α <.05; 2-sided; 1000-sample bootstrapping), with occipital γ power (57-67 Hz; 250-750 milliseconds) and occipital 30- to 50-Hz ITPC (125-200 milliseconds) as dependent variables. Across all groups, γ-band power changes were positively correlated with ITPC values (unstandardized *B*  = 0.077; 95% CI, 0.041-0.113; standardized β coefficient = 0.274; *t*_228_ = 4.3; *P* < .001) and RT variance (*B* = 0.003; 95% CI, 0.001-0.006; β = 0.257; *t*_228_ = 2.7; *P* = .008), and negatively with RTs (*B* = −0.002; 95% CI, −0.004 to −0.001; β = −0.349; *t*_228_ = −3.6; *P* < .001), together explaining 13.2% of variance (*R*^2^ = 0.132). Thirty- to 50-Hz ITPC was positively associated with accuracy (*B* = 0.041; 95% CI, 0.018-0.065; β = 0.198; *t*_228_ = 3.4; *P* = .001) and GAF scores (*B* = 0.036; 95% CI, 0.024-0.047; β = 0.347; *t*_228_ = 5.9; *P* < .001) but negatively correlated with occipital β-band (16-22 Hz) power (*B *= −0.301; 95% CI, −0.562 to −0.040; β = −0.128, *t*_228_ = −2.3; *P* = .02) and CAARMS severity (*B* = −0.025; 95% CI, −0.036 to −0.013; β = −0.281; *t*_204_ = −4.2; *P* < .001) and explained 28.7% of variance (*R*^2^ = 0.287).

### Local and Long-range Oscillations in CHR-P Subgroups

We examined differences between CHR-P subgroups (CAARMS n = 34; SPI-A n = 30; CAARMS/SPI-A n = 55) in MEG activity and behavioral and clinical parameters (eResults, eFigure 6, and eTables 1-3 in the Supplement). Only the combined CAARMS/SPI-A group showed a significant ITPC-deficit relative to HC (TF-cluster approximately 24-72 Hz; approximately 0-300 milliseconds; cluster *P* < .001; 95% CI range, −0.0004 to 0.002). The effect size (*d* = 1.20) was comparable with the FEP group (*d* = 0.93). The CHR-P groups showed no difference in spectral power, while CHR-P individuals with CAARMS and CAARMS/SPI-A criteria showed a selective deficit in long-range connectivity between frontal and occipital cortex (for mSFG-to-IOG connection, see eResults and eFigure 7 in the Supplement).

## Discussion

This study examined neural oscillations during visual processing with a state-of-the art MEG approach to investigate whether emerging psychosis is associated with aberrant oscillatory activity in visual cortex as well as the functional relevance of impaired neural oscillations. Specifically, our data highlight a reduction of phase locking of high-frequency oscillations, a measure of the variability of an ongoing oscillation across trials,^48^ in visual cortices as well as impaired long-range connectivity in CHR-P participants. Importantly, ITPC deficits were also associated with persistent APS, providing important evidence for the role of high-frequency oscillations in clinical staging of emerging psychosis.

Further evidence for the functional relevance of β/γ-band phase locking are significant correlations with RTs, severity of APS, and the combination of SPI-A/CAARMS criteria as well as GAF-scores across participants. In addition, β/γ-band ITPC was associated with induced γ-band power, highlighting the importance of the integrity of early visual processing for large-scale cognition and functioning. These data are consistent with previous findings that have identified associations between compromised sensory processing, impaired functioning, and cognitive deficits in schizophrenia.^50,51,52^

Comparisons between FEP and CHR-P groups revealed overlapping and distinct oscillatory signatures. Induced γ-band oscillations were prominently impaired in the FEP group in visual areas, which was not observed in CHR-P participants. Both groups were characterized by impaired long-range connectivity between visual and frontal cortices while the FEP group also showed reduced visual cortex connectivity. An influential model in schizophrenia has been the disconnectivity hypothesis^36^ as well as the notion of reduced cognitive control mediated by frontal cortices.^53^ Our GC data are consistent with these hypotheses, suggesting a shared feature of both FEP and at-risk participants is the presence of impaired connectivity between sensory regions and frontal cortices.

Impairments in high-frequency oscillations showed a considerable degree of specificity. First, ITPC impairments were only found for activity in the β/γ-band range but not for lower frequencies. Together with the large reductions in induced γ-band activity in the FEP group, these data highlight the unique contribution of high-frequency oscillations toward circuit impairments in emerging psychosis. Second, the CHR-N group showed intact behavioral task parameters as well normal power and phase of high-frequency oscillations in visual cortices and long-range connectivity with only evidence for a circumscribed impairment in local connectivity in visual cortex.

Thus, EEG/MEG readouts could potentially inform clinical decision-making and search for novel treatment opportunities. The search for biomarkers that have prognostic utility and could guide treatments in emerging psychosis is an important objective of research.^22^ This study highlights that impaired γ-band ITPC differentiates between CHR-P individuals who have a high likelihood of persistent APS and transition to psychosis vs CHR-P individuals who show more benign APS. On the other hand, reductions in induced γ-band power emerged as a specific signature of FEP, suggesting that impaired γ-band oscillations could serve as a biomarker for established psychosis that warrants more aggressive treatments, such as antipsychotic medications.

Our observations of increased variability in the timing of β/γ-band oscillations is consistent with formulations that have implicated aberrant glutamatergic and GABAergic neurotransmission as key mechanism for circuit dysfunctions in psychotic disorders.^17,54,55^ Specifically, an increase in variability of neuronal responses can be elicited by *N*-methyl-d-aspartate receptor hypofunction,^56^ suggesting that elevated excitability in sensory regions during the early stages of psychosis may lead to favorable conditions for altered network dynamics to emerge. Moreover, deficits in high-frequency oscillations highlight the contribution of specific GABAergic interneurons, such as parvalbumin or somatostatin-expressing interneurons^13,57^ that are impaired in visual areas in schizophrenia.^58^ In addition to the pharmacologic correction of aberrant circuit dynamics, it is also conceivable that interventions that improve the fidelity of sensory processing through cognitive remediation^59^ or brain stimulation^60^ could potentially prevent the progression of circuit dysfunctions from sensory areas to more extended networks.

### Limitations

This study has several limitations. Although we can predict the persistence of subthreshold psychotic symptoms through MEG data in our CHR-P cohort, further follow-up data are required to test whether abnormalities in high-frequency oscillations can predict transition to psychosis as well as the persistence of Cognitive Disturbances and Cognitive-Perceptive Basic Symptoms criteria. Moreover, these data are only cross-sectional. Accordingly, further studies are required to examine the longitudinal course of oscillatory deficits during emerging psychosis.

## Conclusions

In summary, this advanced MEG analysis provides, to our knowledge, the first comprehensive investigation into the oscillatory signatures during different stages of early psychosis. Specifically, we can show that the timing of high-frequency oscillations in visual cortices is the first impairment to emerge in CHR-P participants in combination with abnormal long-range connectivity. Patients with FEP were characterized by a pronounced reduction in the power of induced γ-band oscillations in combination with reduced β/γ-band ITPC as well as local and long-range connectivity. Importantly, impaired γ-band IPTC-values were associated with the persistence of subthreshold psychotic experiences, suggesting that γ-band oscillations could constitute a possible biomarker for clinical staging of emerging psychosis. Future studies and preclinical research should therefore focus on the circuit-mechanisms mediating precise coordinated neural responses that could offer targets for preventive approaches.

## Notes

Supplement. **eMethods.** Supplementary Methods **eResults.** Supplementary Results **eTable 1.** Overview of Effect Sizes (Cohen’s d) **eTable 2.** Demographic and Clinical Characteristics of CHR-P Subgroups **eTable 3.** Demographic and Clinical Characteristics for Persistent-APS vs Non-Persistent APS CHR-P Subgroups **eFigure 1.** Sensor Data Statistics: Post-Hoc Comparisons **eFigure 2.** Virtual channel TFR/ITPC Group Differences in ROIs **eFigure 3.** Virtual Channel Baseline FFT Spectra over Visual Cortex **eFigure 4.** Virtual Channel ERF Data **eFigure 5.** Correlations MEG data, Behavior and Clinical Parameters **eFigure 6.** Time-Frequency and Inter-Trial-Phase-Coherence in CHR-P subgroups **eFigure 7.** Granger-Causality (GC) connectivity changes in main and CHR-P subgroups
