# Supplementary material for: Association of Magnetoencephalographically Measured High-Frequency Oscillations in Visual Cortex With Circuit Dysfunctions in Local and Large-scale Networks During Emerging Psychosis
Source: JAMA Psychiatry. 2020 Mar 25;77(8):1–12. doi: 10.1001/jamapsychiatry.2020.0284 (PMC7097849; doi:10.1001/jamapsychiatry.2020.0284)
Supplement: Supplement. — eMethods. Supplementary Methods eResults. Supplementary Results eTable 1. Overview of Effect Sizes (Cohen’s d) eTable 2. Demographic and Clinical Characteristics of CHR-P Subgroups eTable 3. Demographic and Clinical Characteristics for Persistent-APS vs Non-Persistent APS CHR-P Subgroups eFigure 1. Sensor Data Statistics: Post-Hoc Comparisons eFigure 2. Virtual channel TFR/ITPC Group Differences in ROIs eFigure 3. Virtual Channel Baseline FFT Spectra over Visual Cortex eFigure 4. Virtual Channel ERF Data eFigure 5. Correlations MEG data, Behavior and Clinical Parameters eFigure 6. Time-Frequency and Inter-Trial-Phase-Coherence in CHR-P subgroups eFigure 7. Granger-Causality (GC) connectivity changes in main and CHR-P subgroups [file jamapsychiatry-77-852-s001.pdf]

Supplementary Online Content

Grent-‘t-Jong T, Gajwani R, Gross J, et al. Association of magnetoencephalographically measured high-frequency oscillations in visual cortex with circuit dysfunctions in local and large-scale networks during emerging psychosis. *JAMA Psychiatry*. Published online March 25, 2020. doi:10.1001/jamapsychiatry.2020.0284

eMethods. Supplementary Method

eResults. Supplementary Results

eTable 1. Overview of Effect Sizes (Cohen’s d)

eTable 2. Demographic and Clinical Characteristics of CHR-P Subgroups

eTable 3. Demographic and Clinical Characteristics for Persistent-APS vs Non-Persistent APS CHR-P Subgroups

eFigure 1. Sensor Data Statistics: Post-Hoc Comparisons

eFigure 2. Virtual channel TFR/ITPC Group Differences in ROIs

eFigure 3. Virtual Channel Baseline FFT Spectra over Visual Cortex

eFigure 4. Virtual Channel ERF Data

eFigure 5. Correlations MEG data, Behavior and Clinical Parameters

eFigure 6. Time-Frequency and Inter-Trial-Phase-Coherence in CHR-P subgroups

eFigure 7. Granger-Causality (GC) connectivity changes in main and CHR-P subgroups

This supplementary material has been provided by the authors to give readers additional information about their work.

© 2020 Grent-‘t-Jong et al. *JAMA Psychiatry.*

eMethods. Supplementary Material

Selection of ROIs, computation of virtual-channel data

Main analyses reported in the main text were based on virtual channel data, reconstructed from 12 regions of interest (ROIs). First, we estimated the individual gamma-band peak frequency at sensors of maximum task response. The mean gamma peak across all 232 participants was determined at 62 ± 5 Hz (Figure 2, Manuscript). As no significant group differences in individual peak frequency were found, we defined induced task-related high-gamma as ranging from 57-67 Hz.

Second, we tested sensor-level data for main group differences in delta (1-3 Hz), theta (4-7 Hz), alpha (8-12 Hz), beta (14-30 Hz), and gamma (57-67 Hz) power, estimated between 250 and 750 ms. We selected this window because there were no influences through evoked responses elicited by the speed-change or stimulus-offset and no contamination of motor-related preparation and execution responses.

Based on a significant main effect of group (cluster of 69 sensors, cluster-$F(3,228) = 341.7$, cluster-$p = 0.0025$) found at sensor-level for gamma-band power (Figure 2A, Manuscript), we then estimated significant locations of whole-head group differences in gamma-band activity (57-67 Hz, 250-750 ms) using DICS beamformers. Source-estimation was performed on a 5 mm grid based on the standard MNI template brain, with the grid linearly warped to individual anatomy. Estimated source activity was computed from the cross-spectral density matrix using normalized lead-fields and common spatial filters for the combined baseline (-500-0 ms) and post-stimulus window of interest (250-750 ms) in order to reduce noise common to both time windows. Induced gamma source activity was expressed as dB change from baseline. A significant main effect of group at source-level ($F(3,228) = 4.1 – 10.0$, $p = 0.0001 – 0.003$) was found in 10 AAL-atlas defined nodes (Figure 2B).

These were then used to guide selection of ROIs for subsequent more fine-grained analyses of virtual channel time-series data. They were computed using LCMV beamformers (Van Veen, van Drongelen et al. 1997), with a regularisation parameter of 20% used to strongly attenuate the amount of leakage from nearby sources. Virtual-channel reconstructed single-trial MEG data were subsequently rescaled, separately per trial and channel, to values between -1 and +1 in order to minimize between-trial variance within participants.

We found this procedure to be the most effective method in compensating for between-trial offset fluctuations, caused by system instabilities, environmental noise and/or participant-induced activity in the < 0.5 Hz range, without losing data samples or introducing artificial changes to the data, for example, associated with the use of more traditional filtering approaches. Internal checks did not indicate any change in topography, timing, or frequency spectrum shift due to this rescaling procedure, but significantly decreased inter-trial variance, as intended, thereby confirming its effectiveness.

Finally, the data from the 12 main ROIs were grouped into clusters for further statistical analyses: 1) Medial superior frontal gyrus (bilateral mSFG), an2) occipital cortex (occROI). The latter was further subdivided into 3 ROIs: a) primary visual cortex (calROI: bilateral calcarine fissure; CAL), b) dorsal-stream areas (dorROI: bilateral cuneus [CUN] and superior occipital gyrus [SOG]), and c) ventral-stream areas (venROI: bilateral middle occipital gyrus [MOG] and inferior occipital gyrus [IOG] (Figure 2C/3C).

MRI-Parameters

Trio Tim scanner: 192 slices, voxel size 1 mm3, FOV=256x256x176 mm3, TR=2250 ms, TE=2.6 ms, FA=9◦.

Cluster-based Permutation Statistics for MEG data

In order to control for the multiple comparison problem (MCP),MEG time-series and frequency domain (virtual) channel analyses applied a cluster-based correction approach. Clusters were defined either by spatiotemporal adjacency (sensor-level analysis: minimally 2 sensors per cluster), adjacent time bins (virtual-channel ERF analyses), adjacent frequency bins (virtual-channel baseline FFT analysis, GC analyses), or time-frequency (TF) bins (virtual-channel TFR and ITPC statistical analyses). Only the statistical analyses of the main group effect of whole-head DICS source estimation of gamma-band activity was False Detection Rate (FDR) corrected for multiple comparisons (For clarity we have plotted in Figure 2B (Manuscript) both corrected [darker red] and uncorrected [lighter red/orange] F-values). In all cases, critical alpha-level was set to 0.05, 2-sided.

© 2020 Grent-‘t-Jong et al. *JAMA Psychiatry.*

Follow-Up Assessments

Participants meeting CHR-P criteria were followed-up for up to 36 months and clinical assessments were conducted at 3, 6, 9, 12, 18, 24, 30 and 36 months using the CAARMS. In addition, the SCID I and SPI-A interviews were administered at 24 and 36 months. Moreover, participants in the CHR-N group were followed up at 3, 6, 12, 18, 24 and 36 months.

Establishing Transition to Psychosis

Transition to psychosis was operationally defined on the CAARMS using the recommended criteria of a global rating scale score of 6 on either unusual thought content, non-bizarre ideas, or disorganized speech, or 5-6 on perceptual abnormalities, with an associated frequency score of 4-6, and with these experiences lasting longer than one week. A SCID DSM-IV Interview was then used to establish the category of psychotic disorder.

Binary Logistic Regression Analyses

MEG and behavioral responses to the visual attention task were used in a Binary Logistic Regression analyses to investigate whether gamma-band ITPC (125-200 ms and 30-50 Hz), response times (RTs), accuracy (ACC), variance in response speed predicted (TR-variability) and persistence of attenuate psychosis symptom (APS) criteria in CHR-P participants at 12 months. We started the initial model selection in an unbiased way by using a backward likelihood ratio selection of the full set of covariates based on 10 ROIs of MEG ITPC data (left and right calcarine, cuneus, superior, middle and inferior occipital gyrus), averaged over 125-200 ms and 30-50 Hz and RTs, ACC and RT-variability. The dependent variable was persistence of APS-criteria at 12 months (Yes [n=39] or No [n=36]). The covariates that significantly contributed to classification accuracy of the APS-persistent group were then used in a reduced model, validated for stability using bootstrapping (n = 5000). Finally, and ROC analysis was performed on the saved predicted probabilities of the final model in order to estimate diagnostic sensitivity and specificity.

Abbreviations and Definitions

ANOVA	Analysis of Variance
APS	Attenuated psychotic symptoms
BACS	Brief Assessment of Cognition in Schizophrenia Battery for Neuropsychological Deficits
CAARMS	Comprehensive Assessment of At Risk Mental States
CAL	Calcarine, a region of interest in visual cortex
CHR-N	CHR-P participants that did not meet CHR-P criteria
CHR-P	Clinical high-risk criteria for psychosis
COPER/COGDIS	Cognitive Disturbances and Cognitive-Perceptive Criteria for Basic Symptoms as defined by the Schizophrenia Proneness Instrument, Adult Version
DAI index	Computed to determine functional direction of Granger-Causality (GC) activity. Formula: (GC-AtoB) – GC(BtoA)) / (GC-AtoB) + (GC-BtoA))

© 2020 Grent-'t-Jong et al. *JAMA Psychiatry.*

	A and B are different nodes. A possible DAI index indicates that the connection between A and B is a feed-forward one, whereas negative values indicate that information flow from area A to B in functionally feedback
DICS	Dynamic Imaging of Coherent Sources
ECG	Electrocardiogram
Evoked activity	EEG/MEG activity that is phase- and time-locked to stimulus onset, in part captured by early event-related field activity (up to ~200-250 ms) with contributions of frequencies up to about 20-30 Hz, but also by inter-trial phase coherence (ITPC), which captures also higher-frequency (>~30 Hz) evoked activity
E/I-Balance	Balance between Excitation and Inhibition
EEG/MEG	Electro/Magnetoencephalography
ERFs	Event Related Fields are the MEG-analog to Event-Related Potentials
FB	Feedback
FF	Feedforward
FFT	Fast-Fourier Transformed
FEP	First-episode psychosis
GAF	Global Assessment of Functioning
GABA	gamma-Aminobutyric Acid
HC	Healthy control participants
ICA	Independent Component Analysis
Induced activity	EEG/MEG activity that is time-locked, but not phase-locked to the stimulus, typically investigated in the frequency domain (e.g. gamma power)
IOG	Inferior occipital gyrus, a region of interest in visual cortex
ITPC	Inter-Trial Phase Coherence is a measure of the consistency of the phase-angle across single channels/sensors across trials
LCUN	Left Cuneus, a region of interest in visual cortex
LMOG	Left middle occipital gyrus, a region of interest in visual cortex
MCP	Multiple Comparison Problem
MSFG	Medial superior frontal gyrus, a region of interest in frontal cortex
PANSS	Positive and Negative Symptom Scale
RCUN	Right Cuneus, a region of interest in visual cortex
ROC	Receiver Operating Characteristic Curve

© 2020 Grent-'t-Jong et al. *JAMA Psychiatry.*

ROIs	Regions-Of-Interest
RTs	Reaction Times
SCID	Structured Clinical Interview for the DSM-IV
ScZ	Schizophrenia
SOG	Superior occipital gyrus, a region of interest in visual cortex
SQUID	Superconducting Quantum Interference Device is a very sensitive magnetometer used in MEG
SPI-A	Schizophrenia Proneness Instrument, Adult version
TF	Time-Frequency
TFR	Time-Frequency Responses
YouR	Youth Mental Health Risk and Resilience (YouR) Study
Virtual Channel	Virtual channels are derived from source-localization approaches that display the time-resolved signal of these sources

© 2020 Grent-'t-Jong et al. *JAMA Psychiatry.*

eResults. Supplementary Results

Clinical Outcomes in CHR-P

Analyses included CHR-P participants who completed MEG-baseline measurements, regardless of follow-up period and completion of assessments. Of the 119 CHR-P participants, follow-up information was available for 104 after baseline MEG-measurement with mean follow-up period of 18.2 months. Overall, of the 119 CHR-P participants, 12% had completed the entire follow-up period (36 months), 34% up to 24 months and 62% up to 12 months. Last clinical follow-up assessment was at 3 months for 3 (2.9%) CHR participants, at 6 months for 14 (13.5%), at 9 months for 6 (5.8%), at 12 months for 23 (22.1%), at 15 months for 1 (1.0%), at 18 months for 15 (14.4%), at 24 months for 24 (23.1%), at 30 months for 6 (5.8%) and finally at 36 month for 12 participants (11.5%). Accordingly, it is conceivable that the CHR-P group may contain participants who eventually transition to psychosis because they did not return for follow-up assessment or because they have not reached the 36-month assessment yet. Overall, 9 CHR-P participants converted to psychosis (mean conversion period: 17.3 months).

Predicting Clinical Outcomes from MEG data

ITPC data (30-50 Hz, 125-200 ms) from 10 occipital ROIs, together with RTs, accuracy, variability of RTs were entered into an unbiased binary logistic regression model, using a backward likelihood ratio method to find the set of covariates that significantly predicted persistence of APS-criteria in the CHR-P group. The results of this initial model showed that only ITPC values from three occipital cortex ROIs (LCUN, RCUN, and LMOG) contributed significantly to the model (omnibus χ^2 (3) = 14.4, p = 0.002; variables in the equation, LCUN: Wald χ^2 (1) = 7.1, p = 0.008, Exp(B) = 2.74; RCUN: Wald χ^2 (1) = 5.2, p = 0.023, Exp(B) = 0.50; LMOG: Wald χ^2 (1) = 6.4, p = 0.011, Exp(B) = 0.43). Together, these predictors explained 22.2% of total variance (Nagelkerke R^2= 0.222) and accurately classified 75.0% of the cases in the APS persistent and 71.8% in the APS non-persistent group (overall 73.3%). This discrimination performance was confirmed by a significant (p = 0.001) ROC Curve analysis (Area Under Curve of 0.728). Finally, bootstrapping (n = 5000) to validate the reduced model revealed that the model was very stable (p-value improved slightly to 0.007 for LCUN, 0.022 for RCUN, and 0.008 for LMOG).

Results CHR-P subgroup analyses

CHR-P participants (n=119) were further subdivided into three groups to examine differences in clinical and MEG-parameters: 1) SPI-A (n = 30), 2) CAARMS (n = 34), and 3) CAARMS/SPI-A (n = 55). Subsequently, we tested for between-subgroup and subgroup versus control differences in ITPC, TFR, or Granger-Causality changes (see eTable 1 and 2, eFigure 6 and eFigure7).

No differences were found for power changes across occipital ROIs (within 1-90 Hz, 0-750 ms) in any CHR-P subgroup contrast. However, ITPC responses across occipital ROIs were significantly reduced in the CAARMS/SPI-A group compared to HC (Time-Frequency cluster ~24-72 Hz, ~0-300ms, cluster-t(102) = -622.2, cluster-p < 0.001) with comparable effect sizes as in the FEP group (effect-sizes: FEP, d = 0.93, CAARMS/SPI-A, d = 1.20). Additional testing focusing on peak ITPC activity only (averaged over 30-50 Hz, 125-200 ms and all 10 occROIs; see eFigure6, bottom row left figures) revealed an additional significant effect for the SPI-A group compared to HC (t(77) = -2.3, p = 0.029, d = 0.58). Further reduction of these analyses to the three ROIs (RCUN, LCUN, LMOG) that were predictive of one-year persistence of APS-criteria revealed that the ITPC activity in the 30-50 Hz range in the CAARMS/SPI-A group was also significant impaired compared to the CAARMS group (t(87) = -2.3, p = 0.020).

Finally, GC changes were tested across the three connections (eFigure7) showing main group effects: CAL-to-SOG (50-77 Hz), IOG-toCAL (1-14 Hz) and mSFG-to-IOG (24-41 Hz). Compared to controls, the CAL-to-SOG connectivity was significantly lower in the CAARMS group (t(81) = -2.0, p = 0.046). The SPI-A group showed significantly decreased IOG-to-CAL (t(77) = -2.2, p = 0.004) and significantly increased mSFG-to-IOG connectivity (t(77) = 3.1, p = 0.004). Finally, the mSFG-to-IOG connection was also significantly upregulated in the CAARMS/SPI-A group (t(102) = 2.8, p =0.009).

Comparisons APS-persistent vs. APS Non-Persistent CHR-P

Thirty-nine CHR-P participants continued to meet APS-criteria (APS-persistent group) while 36 CHR-P participants were characterized by a remission of APS-criteria. Compared to the non-persistent group, the APS persistent group had

© 2020 Grent-'t-Jong et al. *JAMA Psychiatry.*

significantly higher CAARMS severity scores (p=0.014)) and significantly lower Global Role Functioning scores (GF-role: p= 0.037). Furthermore, 9 out of 119 CHR-P participants made a transition to psychosis (mean follow-up period: ~17.3 months). 8 transitions occurred in the APS-persistent group.

eFigure 1. Sensor Data Statistics: Post-hoc Comparisons

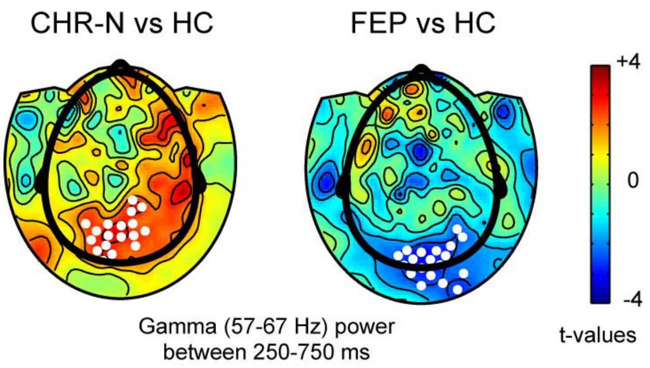

The main group effect for gamma-band power (57-67 Hz, 250-750 ms) was followed up by pairwise comparisons between groups, using non-parametric Monte-Carlo based permutation independent-sample t-tests, cluster-corrected for multiple comparisons at alpha-level 0.05, 2-sided. These analyses indicated increased gamma power for CHR-N (cluster-t(85) = 48.4, cluster-p = 0.032) over superior occipital and right parietal sensors (left figure above: white dots indicate significant sensors) and decreased power (cluster-t(73) = -50.7, cluster-p = 0.0210) for FEP (right figure above), compared to controls, over medial and inferior- and middle occipital sensors.

© 2020 Grent-'t-Jong et al. *JAMA Psychiatry.*

eFigure 2. Virtual channel TFR/ITPC Group Differences

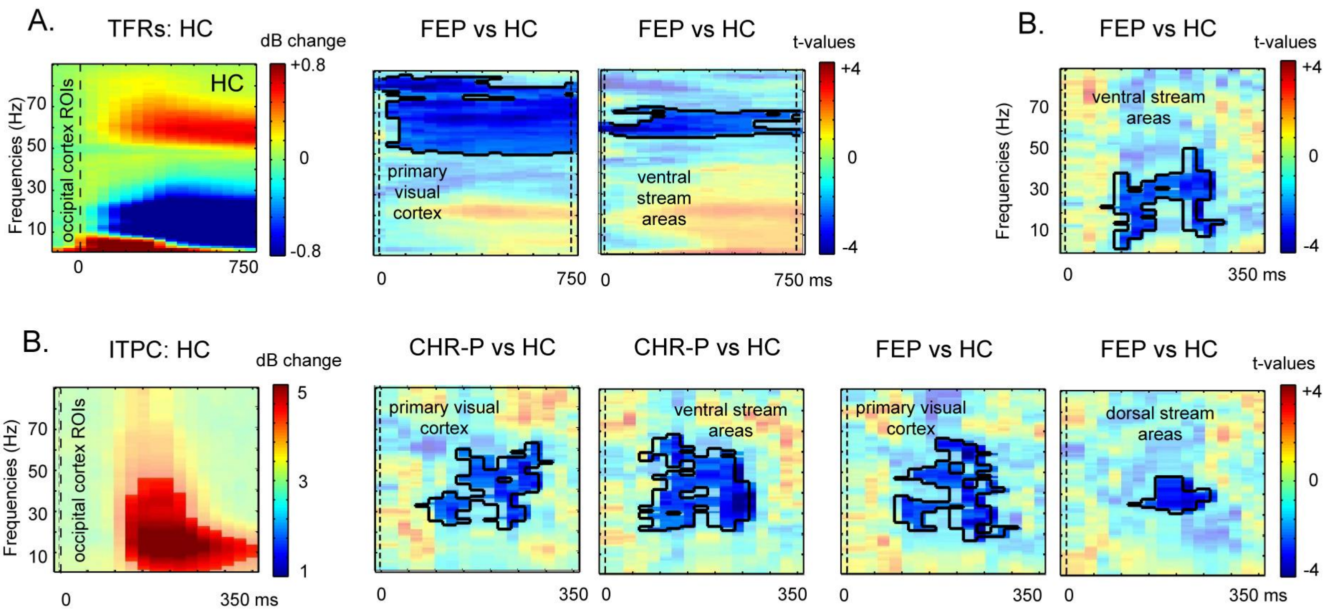

A. Differences in FEP group, compared to controls (HC), in primary visual cortex and ventral stream ROIs with significant clusters outlined and the remaining non-significant time-frequency bins masked out (opacity 0.45). **B.** As panel A, but for ITPC responses in CHR-P and FEP groups, compared to HC.

Significant TFR-ranges and cluster t/p-values for all sub-ROI results:

FEP – HC	calROI	0-750 ms	50-88 Hz	$t(73) = -113.4$	$p < 0.001$
FEP – HC	venROI	0-750 ms	58-72 Hz	$t(73) = -421.7$	$p = 0.036$

Significant ITPC-ranges and cluster t/p-values for all sub-ROI results:

CHR-P – HC	calROI	75-300 ms	22-63 Hz	$t(166) = -385.5$	$p = 0.002$
CHR-P – HC	venROI	75-250 ms	22-69 Hz	$t(166) = -604.6$	$p < 0.001$
FEP – HC	calROI	100-300 ms	17-66 Hz	$t(73) = -480.5$	$p < 0.001$
FEP – HC	dorROI	125-250 ms	31-48 Hz	$t(73) = -153.2$	$p = 0.034$
FEP – HC	venROI	75-275 ms	2-52 Hz	$t(73) = -415.6$	$p < 0.001$

eFigure 3. Virtual Channel Baseline FFT Spectra Over Visual Cortex

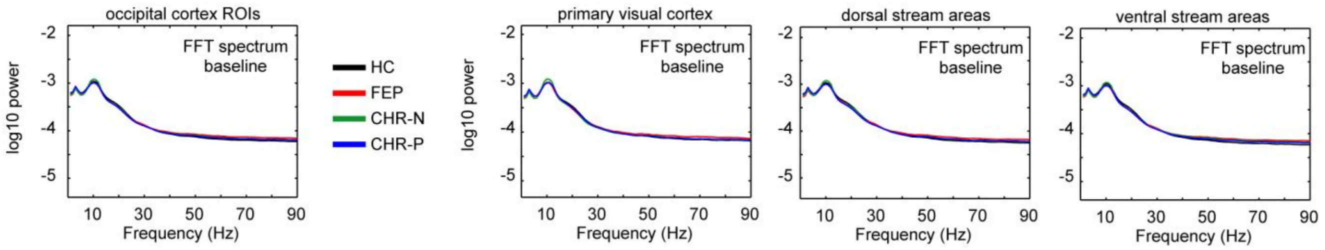

Left panel shows baseline spectra for all groups, averaged over all occipital ROIs. The three panels on the right show the spectra for ROIs in the primary visual cortex, dorsal and ventral stream. No differences were found between groups in any ROI or frequency bin.

© 2020 Grent-'t-Jong et al. *JAMA Psychiatry.*

eFigure 4. Event-Related Field (ERF) Responses

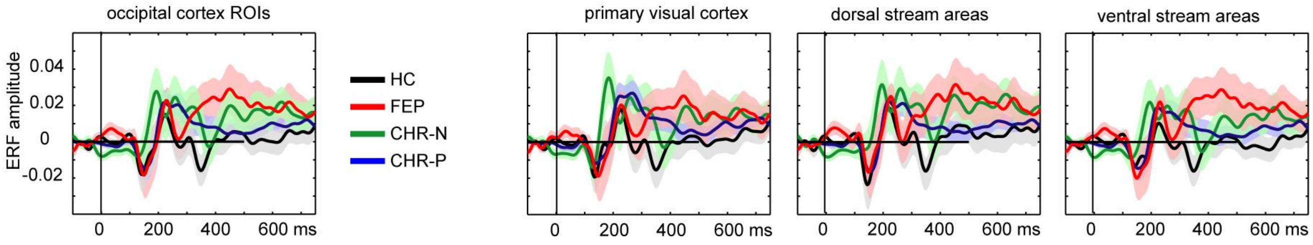

Left panel shows ERFs for all groups (error-bars indicate standard error of the mean), averaged over all occipital ROIs. The three panels on the right show the ERFs for ROIs in the primary visual cortex, dorsal and ventral stream. No group differences were found for any time window up to 750 ms.

© 2020 Grent-‘t-Jong et al. *JAMA Psychiatry.*

eFigure 5. Correlations MEG data, Behavior and Clinical parameters

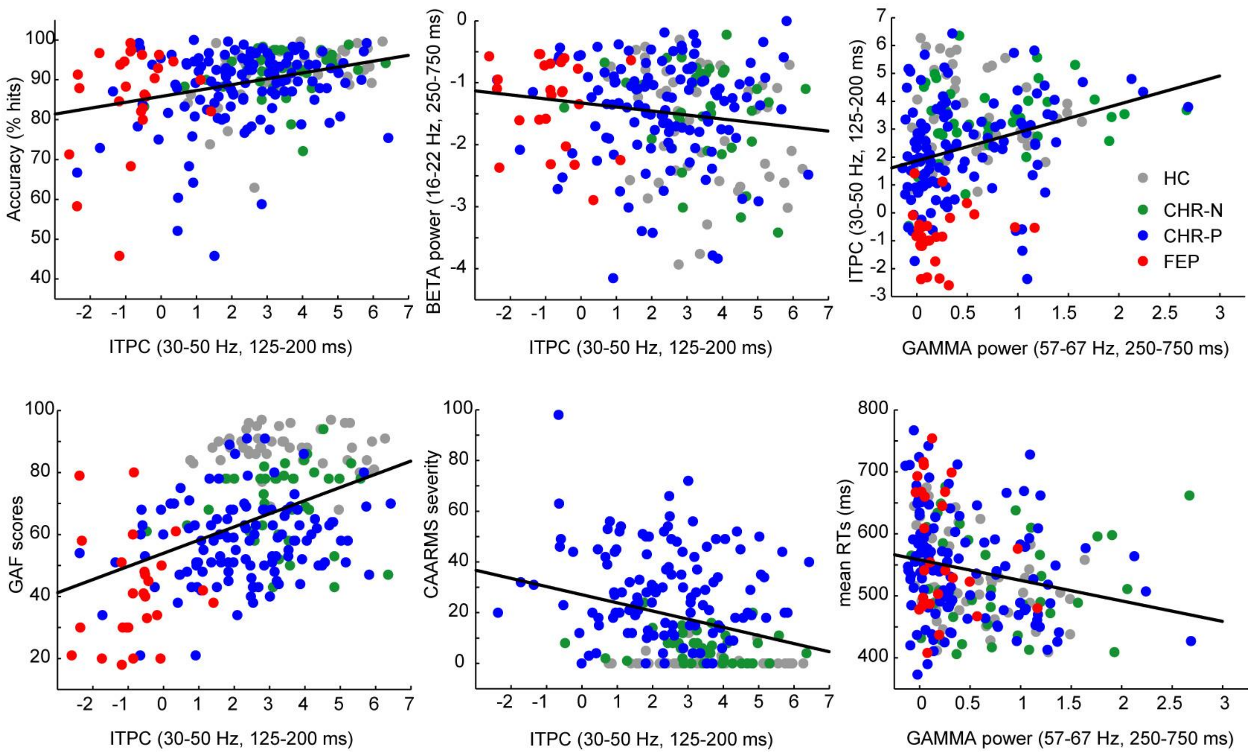

Scatterplots showing significant correlations for occipital ROIs mean ITPC values (30-50 Hz, 125-200 ms) and mean occipital Gamma power (57-67 Hz, 250-750 ms). Gamma-band power correlated negatively with RTs, and positively with ITPC values, but not with clinical data. In contrast, ITPC correlated negatively with beta power and CAARMS-severity, while positive correlations were observed with response accuracy and GAF scores.

© 2020 Grent-‘t-Jong et al. *JAMA Psychiatry.*

eFigure 6. Time-frequency power and Inter-Trial-Phase-Coherence changes in CHR-P Subgroups

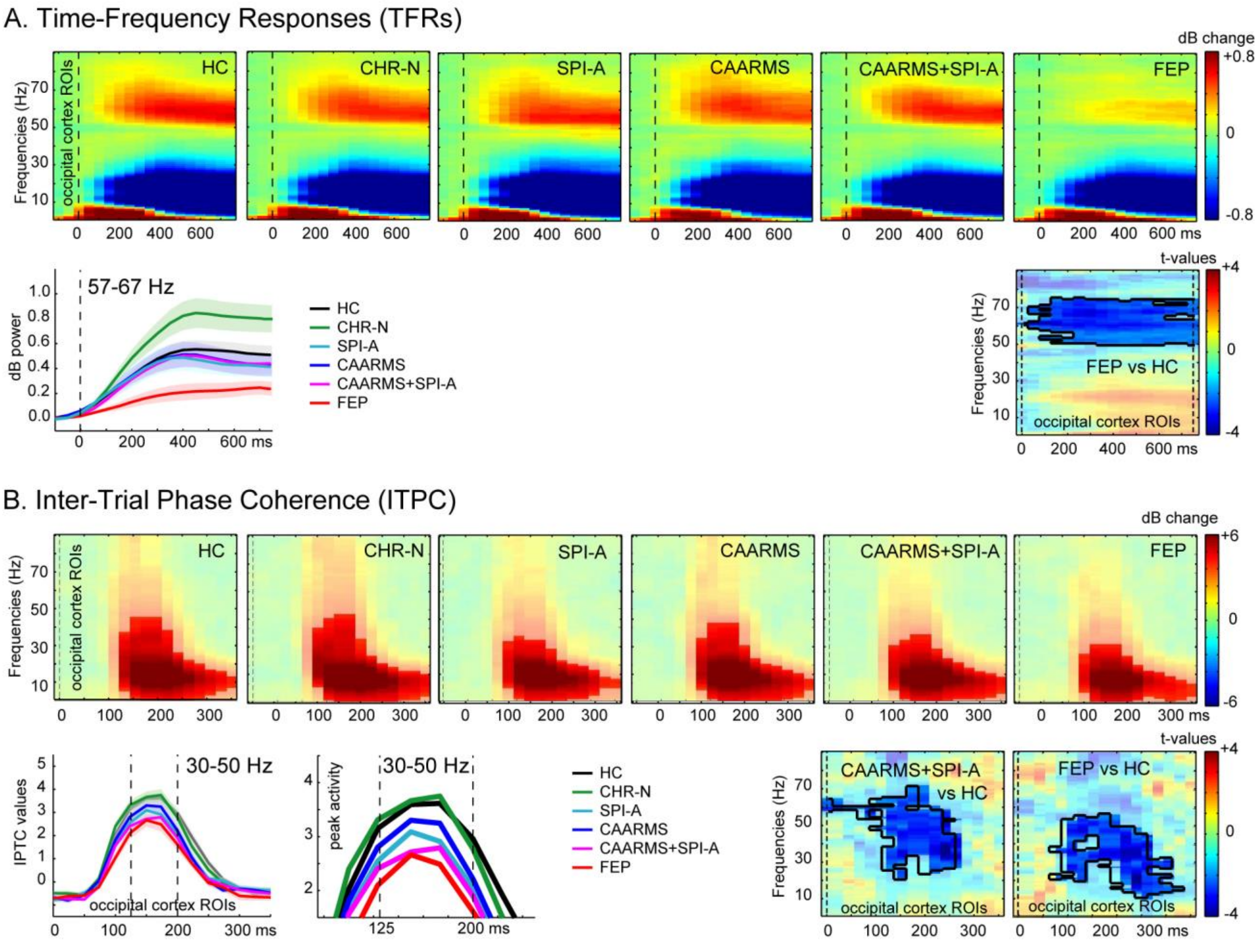

Time-Frequency Responses (TFR plots) for HC group (n=49), CHR-N (n=38), CHR-P subgroups (SPIA, (n=30), CAARMS (n=34) and CAARMS/SPI-A (n=55) – and FEP patients (n=26). Data were averaged over 10 occipital ROIs (left/right calcarine, cuneus, inferior, middle, and superior occipital gyrus). B) As in A but for Inter-Trial-Phase-Coherence (ITPC)-

© 2020 Grent-'t-Jong et al. *JAMA Psychiatry.*

eFigure 7. Granger-Causality (GC) connectivity changes in main and CHR-P subgroups

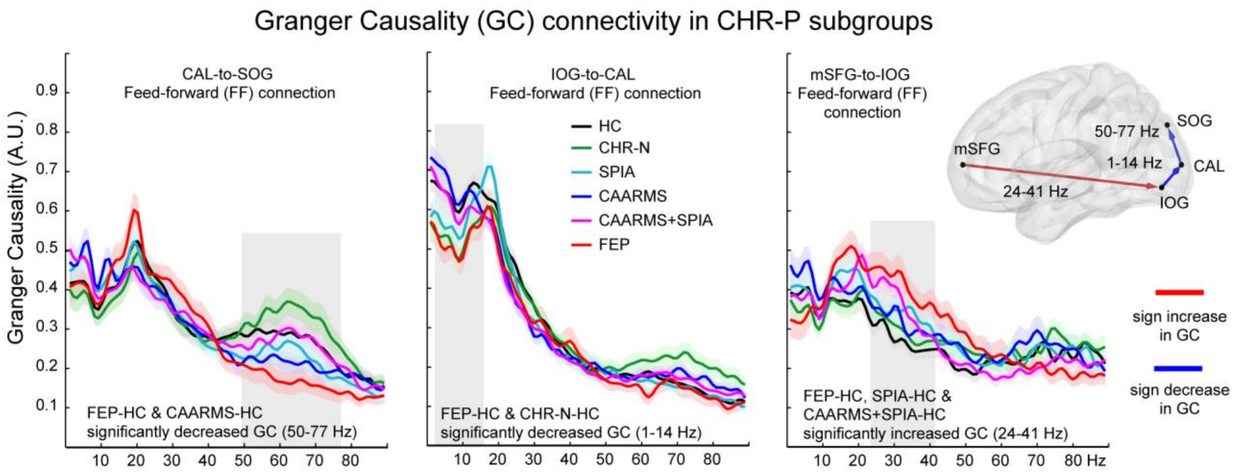

Results of cluster-based statistics on Granger Causality (GC) data, showing range of significant effects in the main groups (FEP, CHR-P, and CHR-N) and CHR-P subgroups (SPI-A, CAARMS, and CAARMS/SPI-A), compared to HC. The main connections tested are plotted on a smoothed surface of a standard MNI brain, with red lines representing increased and blue lines decreased GC values, compared to HC. For each significant connection, GC values are plotted across the frequency spectrum, separately per group (with error bars indicating standard error of the mean), and a horizontal line indicating the frequency range of significant group effects. GC was computed for data between 250-750 ms, post-stimulus onset. DAI indices were all positive in the significant contrasts, indicating feed-forward flow of information between the nodes.

eTable 1. Overview of Effect Sizes (Cohen's d)

CHR-N vs HC	Effect size	CHR-P vs HC	Effect size	FEP vs HC	Effect size
BEHAVIORAL PERFORMANCE					
--	--	⇓ ACC	0.50	--	--
--	--	--	--	⇑ VRT	0.70
INTER-TRIAL PHASE COHERENCE					
--	--	⇓ occROI	0.63	⇓ occROI	0.93
--	--	⇓ calROI	0.64	⇓ calROI	1.13
--	--	⇓ venROI	0.69	⇓ venROI	0.80
--	--	--	--	⇓ dorROI	0.85
GAMMA POWER					
--	--	--	--	⇓ occROI	1.14
--	--	--	--	⇓ calROI	1.26
--	--	--	--	⇓ venROI	1.10
--	--	--	--	⇓ dorROI	0.90
GRANGER CAUSALITY [a]					
--	--	--	--	⇑ CAL2SOG (FF)	0.74
--	--	⇑ mSFG2IOG (FF)	0.54	⇑ mSFG2IOG (FF)	1.04
⇑ IOG2CAL (FB)	0.73	--	--	⇑ IOG2CAL (FB)	0.84

Abbreviations: ACC = response accuracy, VRT = response time variance, occROI = all 10 occipital ROIs (calROI, dorROI, venROI included), calROI = primary visual cortex ROIs (calcarine fissure), dorROI = dorsal stream areas (cuneus, superior occipital gyrus), venROI = ventral stream ROIs (inferior and middle occipital gyrus), FF = feed-forward connection, FB = feedback connection, CAL2SOG = calcarine fissure to superior occipital gyrus, mSFG2IOG = medial superior frontal gyrus to inferior occipital gyrus, IOG2CAL = inferior occipital gyrus to calcarine fissure. Arrows indicate direction of effect.

[a] Granger Causality effect-sizes were computed across significant frequency bins within a (set of) connected areas.

ADDITIONAL effect sizes for different UHR subgroups:

ITPC in occROI	CAARMS/SPI-A	d = 1.20
GC for CAL2SOG	CAARMS	d = 0.45
GC for mSFG2IOG	SPI-A	d = 0.73
	CAARMS/SPI-A	d = 0.55
Response Accuracy	CAARMS/SPI-A	d = 0.43

© 2020 Grent-'t-Jong et al. *JAMA Psychiatry.*

eTable 2. Demographic and Clinical Characteristics of CHR-P Subgroups

	SPI-A	CAARMS	CAARMS/ SPI-A	Pairwise comparisons [a]
**Number of participants**	30	34	55	
**Age**, years (SD)	22 (4.1)	21 (4.0)	22 (4.8)	--
**Sex**, male/female (%male)	10/20 (33.4)	6/28 (17.6)	16/39 (29.1)	--
**Education**, years (SD)	15 (2.8)	14 (2.9)	16 (3.3)	SPI-A – HC : p = 0.002
**BACS** [b], mean (SD)				
Verbal memory	-0.19 (1.4)	-0.40 (1.3)	-0.43 (1.2)	--
Digit sequencing	0.22 (1.1)	-0.30 (1.7)	-0.27 (1.5)	--
Token motor	-0.95 (1.6)	-1.09 (1.2)	-0.93 (1.2)	SPI-A – HC : p = 0.027
				CAARMS – HC : p < 0.001
				CAARMS/SPI-A – HC : p < 0.001
Verbal fluency	-0.24 (0.9)	-0.13 (1.5)	0.09 (1.3)	--
Symbol coding	-0.35 (1.4)	-0.75 (1.2)	-0.61 (1.0)	CAARMS – HC : p = 0.017
				CAARMS/SPI-A – HC : p = 0.012
Tower of London	0.01 (1.5)	-0.65 (1.4)	-0.06 (1.4)	
Composite score	-0.39 (1.3)	-0.91 (1.7)	-0.58 (1.2)	CAARMS – HC: p = 0.034
				CAARMS/SPI-A – HC: p = 0.042
**CAARMS**, mean (SD)				
Unusual Thought Content	1 (1.8)	1 (1.7)	2 (2.0)	
Non-bizarre Ideas	2 (1.8)	3 (1.7)	3 (1.7)	
Perceptual Abnormalities	2 (1.6)	3 (1.6)	3 (1.3)	
Disorganized Speech	1 (1.3)	1 (1.2)	2 (1.4)	
Total severity score	15 (13.5)	27 (13.9)	38 (16.6)	SPI-A – CAARMS : p = 0.004
				SPI-A – CAARMS/SPI-A: p < 0.001
				CAARMS – CAARMS/SPI-A: p = 0.004
**Transitions (%)**	0	4 (11.7%)	5 (9.9%)	
**GAF**, mean (SD)	63 (13.3)	58 (12.8)	54 (12.9)	SPIA – HC : p < 0.001
				CAARMS – HC : p < 0.001
				CAARMS/SPI-A – HC : p < 0.001
				SPI-A – CAARMS/SPI-A: p = 0.012
**GF-role**, mean (SD)	7.7 (1.3)	7.6 (0.9)	7.1 (1.3)	SPI-A – HC : p = 0.010
				CAARMS – HC : p < 0.001
				CAARMS/SP-IA – HC : p < 0.001
**GF-social**, mean (SD)	7.8 (1.4)	7.5 (1.0)	7.3 (1.3)	SPI-A – HC : p = 0.002
				CAARMS – HC : p < 0.001
				CAARMS/SPI-A – HC : p < 0.001
**Medication** [c]				
None	15	19	25	
Anti-depressants	12	9	25	
Mood stabilizers	0	1	1	
Anti-Psychotics	0	2	1	
Other (Unknown)	3 (0)	3 (0)	3 (0)	
**COGDIS/COPER/both items**	4 / 15 / 11	-	9 / 22 / 24	
**MINI categories**				
Depressive/Mood disorders	19	19	37	
Anxiety disorders/PTSD/OCD	18	27	42	
Drug/alcohol abuse/dependence	10	9	23	
Eating Disorders	0	3	7	
**Task Performance**				
Accuracy (% correct, SD)	89.3 (6.1)	86.0 (11.7)	88.5 (10.1)	CAARMS/SPI-A – HC : p = 0.036
Mean response time (ms, SD)	529 (66.9)	566 (80.7)	541 (94.6)	--
Response variance [d] (ms, SD)	159.4 (36.3)	177.8 (43.0)	158.6 (43.5)	--

© 2020 Grent-‘t-Jong et al. *JAMA Psychiatry.*

Abbreviations: SD = standard deviation of the mean, ms = milliseconds

[a] F-tests are Welch based; alpha=0.05, 2-sided, 1000 samples bootstrapping, reported post-hoc tests are Games-Howell correction for Type I errors

[b] BACS scores for clinical groups were standardized to control group data, controlled for sex category

[c] If multiple medications were reported, they were scored separately in the different categories listed

[d] Response variance = standard deviation of response times across trials

© 2020 Grent-‘t-Jong et al. *JAMA Psychiatry.*

eTable 3. Demographic and Clinical Characteristics for APS persistent vs APS non-persistent CHR-P subgroups

	**APS persistent**	**APS non-persistent**	**Pairwise comparisons** [a]
**Number of participants**	39	36	
**Age**, years (SD)	22 (5.0)	22 (4.1)	
**Sex**, male/female (%male)	10/29 (25.7)	7/29 (19.4)	
**Education**, years (SD)	15 (3.5)	15 (2.6)	
**BACS** [b], mean (SD)			
Verbal memory	-0.52 (1.1)	-0.20 (1.3)	
Digit sequencing	-0.50 (1.8)	-0.05 (1.5)	
Token motor	-1.02 (1.4)	-0.95 (1.1)	APS-persistent – HC : p < 0.001
			APS-non-persistent – HC : p < 0.001
Verbal fluency	0.00 (1.4)	0.11 (1.4)	
Symbol coding	-0.67 (1.0)	-0.57 (1.2)	APS-persistent – HC : p = 0.005
Tower of London	-0.21 (1.4)	-0.35 (1.6)	
Composite score	-0.78 (1.4)	-0.55 (1.6)	APS-persistent – HC : p = 0.01
**CAARMS**, mean (SD)			
Unusual Thought Content	2 (2.0)	1 (1.9)	
Non-bizarre Ideas	3 (1.5)	3 (1.9)	
Perceptual Abnormalities	3 (1.5)	3 (1.5)	
Disorganized Speech	1 (1.4)	1 (1.4)	
Total severity score	37 (14.2)	30 (15.1)	APS-persistent – APS-non-persistent : p = 0.014 [e]
**GAF**, mean (SD)	53 (11.5)	58 (13.1)	APS-persistent – HC : p < 0.001
			APS-non-persistent – HC : p < 0.001
**GF-role**, mean (SD)	7.0 (1.1)	7.6 (1.1)	APS-persistent – HC : p < 0.001
			APS-non-persistent – HC : p < 0.001
			APS-persistent – APS-non-persistent : p = 0.037
**GF-social**, mean (SD)	7.2 (1.1)	7.5 (1.1)	APS-persistent – HC : p < 0.001
			APS-non-persistent – HC : p < 0.001
**Medication** [c]			
None	21	18	
Anti-depressants	13	14	
Mood stabilizers	0	2	
Anti-Psychotics	0	1	
Other (Unknown)	5 (0)	1 (0)	
**CHR-P categories**			
SPI-A only (COGDIS/COPER/both items)	-	-	
CAARMS only	11	19	
CAARMS + SPI-A (COGDIS/COPER/both items)	28 (5 / 16 /7)	17 (4 / 6 /7)	
**Transitions (%)**	8 (20.5%)	0	
**MINI categories**			
Depressive/Mood disorders	25	21	
Anxiety disorders/PTSD/OCD	32	29	
Drug/alcohol abuse/dependence	17	12	
Eating Disorders	6	4	
**Task Performance**			
Accuracy (% correct, SD)	87.1 (11.2)	87.5 (11.5)	APS-persistent – HC : p = 0.041
Mean response time (ms, SD)	541 (66.4)	535 (103.2)	
Response variance [d] (ms, SD)	168.6 (42.0)	159.4 (49.3)	

© 2020 Grent-‘t-Jong et al. *JAMA Psychiatry.*

Abbreviations: SD = standard deviation of the mean, ms = milliseconds

[a] F-tests are Welch based; alpha=0.05, 2-sided, 1000 samples bootstrapping, reported post-hoc tests are Games-Howell correction for Type I errors

[b] BACS scores for clinical groups were standardized to control group data, controlled for sex category

[c] If multiple medications were reported, they were scored separately in the different categories listed

[d] Response variance = standard deviation of response times across trials

e Direct main effect between CAARMS persistence groups

References

Van Veen, B. D., et al. (1997). "Localization of brain electrical activity via linearly constrained minimum variance spatial filtering." IEEE Trans Biomed Eng **44**(9): 867-880.

© 2020 Grent-‘t-Jong et al. *JAMA Psychiatry.*
